# FoxM1-dependent RAD51 and BRCA2 signaling protects idiopathic pulmonary fibrosis fibroblasts from radiation-induced cell death

**DOI:** 10.1038/s41419-018-0652-4

**Published:** 2018-05-22

**Authors:** Jintaek Im, Jessica Lawrence, Davis Seelig, Richard S. Nho

**Affiliations:** 1 Division of Pulmonary, Allergy, Critical Care, and Sleep Medicine, Department of Medicine, University of Minnesota, 420 Delaware SE, Minneapolis, MN 55455 USA; 2 Department of Veterinary Clinical Sciences, College of Veterinary Medicine & Masonic Cancer Center, University of Minnesota, St. Paul, MN 55108 USA

## Abstract

Radiation therapy is critical for the control of many tumors and lung is an important dose-limiting organ that impacts radiation dose prescribed to avoid irreversible pulmonary fibrosis in cancer survivors. Idiopathic pulmonary fibrosis (IPF) is a chronic, irreversible lung disease caused by aberrantly activated lung (myo)fibroblasts. The presence of pro-fibrotic, apoptosis-resistant fibroblasts in IPF promotes progressive fibrosis and may have a role in other diseases, if these resistant cells are selected for as a consequence of treatment. However, the pathological response of IPF fibroblasts to radiation compared to non-IPF lung fibroblasts is not known. To address this, we examined fibroblast viability following radiation in lung fibroblasts from IPF and non-IPF patients and the underlying mechanism that protects IPF fibroblasts from radiation-induced death. IPF fibroblasts are significantly more resistant to apoptosis compared to non-IPF lung fibroblasts, suggesting that resistance to radiation-induced cell death is a predominant mechanism leading to lung fibrosis. Analysis of γH2AX induction demonstrated that radiation-induced DNA damage is reduced in IPF fibroblasts and correlates to the activation of the transcription factor forkhead box M1 (FoxM1) and subsequent upregulation of DNA repair proteins RAD51 and BRCA2. FoxM1 activation occurs secondary to FoxO3a suppression in IPF fibroblasts while restoration of FoxO3a function sensitizes IPF fibroblasts to radiation-induced cell death and downregulates FoxM1, RAD51, and BRCA2. Our findings support that increased FoxO3a/FoxM1-dependent DNA repair may be integral to the preservation of death-resistant fibrotic fibroblasts after radiation and that selective targeting of radioresistant fibroblasts may mitigate fibrosis.

## Introduction

Radiation therapy is prescribed in over 50% of patients receiving cancer treatment. Radiation-induced toxicities are relatively common following radiation when normal lung is in close proximity to tumor. While pneumonitis is an early and potentially reversible toxicity, pulmonary fibrosis is delayed, progressive and can impair normal lung function^1,2^. Rates of pulmonary fibrosis can be as high as 70–80% in high dose regions of irradiated lung^3^. It is currently unclear whether radiation-induced lung fibrosis (RILF) results from failure of normal healing after pneumonitis or is a separate, complicating event^4,5^. Thus, it is difficult to predict the true risk of RILF, for which there are no effective treatment strategies^2,4,6,7^. Recent work in idiopathic pulmonary fibrosis (IPF), a progressive and lethal lung disease, showed that fibroblasts derived from IPF patients maintain an apoptosis-resistant phenotype in response to various stressors^8–14^. Elucidation of this mechanism is crucial in understanding fibrotic disease, regardless of the inciting cause.

Ionizing radiation initially injures pulmonary epithelial cells, releasing pro-inflammatory cytokines that recruit inflammatory cells^15^. Fibroblasts become activated and produce collagen-rich extracellular matrix during repair of basement membranes^15^. Contrary to normal healing, thoracic radiation inappropriately activates myofibroblasts, which promote the deposition of type I collagen that destroys parenchyma and establishes a niche for ongoing fibrosis^16,17^. To further compound this injury, alveolar epithelial cells may undergo trans-differentiation into myofibroblasts in IPF and RILF^15,18^. The most lethal event following radiation to non-hematopoietic cells is the induction of DNA double-strand breaks (DSB), which induces mitotic catastrophe and apoptosis after 2–6 days^19–23^. Under normal physiologic conditions, DNA DSB trigger a cascade of events that encourage repair at the site of DNA damage^24^. Homologous recombination (HR) repair following DNA DSB is a primary, high-fidelity mechanism of radiation repair in human cells. An important step in HR is recruitment of the repair protein RAD51 by breast cancer-associated gene 2 (BRCA2) to the damaged DNA sites; the alteration of these proteins renders cells resistant to cytotoxic damage^25,26^. FoxM1, a member of the Forkhead family of transcription factors, is known to upregulate DNA repair proteins such as RAD51 and BRCA2, thereby protecting cells from radiation-induced DNA damage^27,28^. FoxM1 was increased in irradiated murine lung tissue and in human IPF fibrotic lesions; moreover, the conditional deletion of FoxM1 prevented lung fibrosis^29^. FoxM1 activation occurs following suppression of FoxO3a, indicating a negative feedback loop exists between these two family members^27,30^, and FoxO3a is aberrantly suppressed in IPF fibroblasts and patient IPF lung tissues^8,9,31,32^. We therefore sought to examine the FoxO3a/FoxM1-dependent pathway in IPF cells in response to ionizing radiation. We found that IPF fibroblasts sustain less radiation-induced DNA damage compared to non-IPF lung fibroblasts and are highly viable after radiation. We further demonstrated that enhanced FoxM1 in IPF fibroblasts leads to increased RAD51 and BRCA2 gene expression. Following FoxO3a over-expression or FoxM1 silencing, RAD51 and BRCA2 expression is abrogated and sensitizes IPF fibroblasts to radiation-induced death. We propose that FoxM1 may be pivotal in selecting apoptosis-resistant fibrotic fibroblasts within irradiated lung tissue, contributing to the development and progression of RILF. FoxM1 may therefore represent a therapeutic target as it regulates fibroblast survival following radiation damage.

## Results

### IPF fibroblasts are highly viable and resistant to radiation- or bleomycin-induced cell death on collagen

**Fig. 1:**
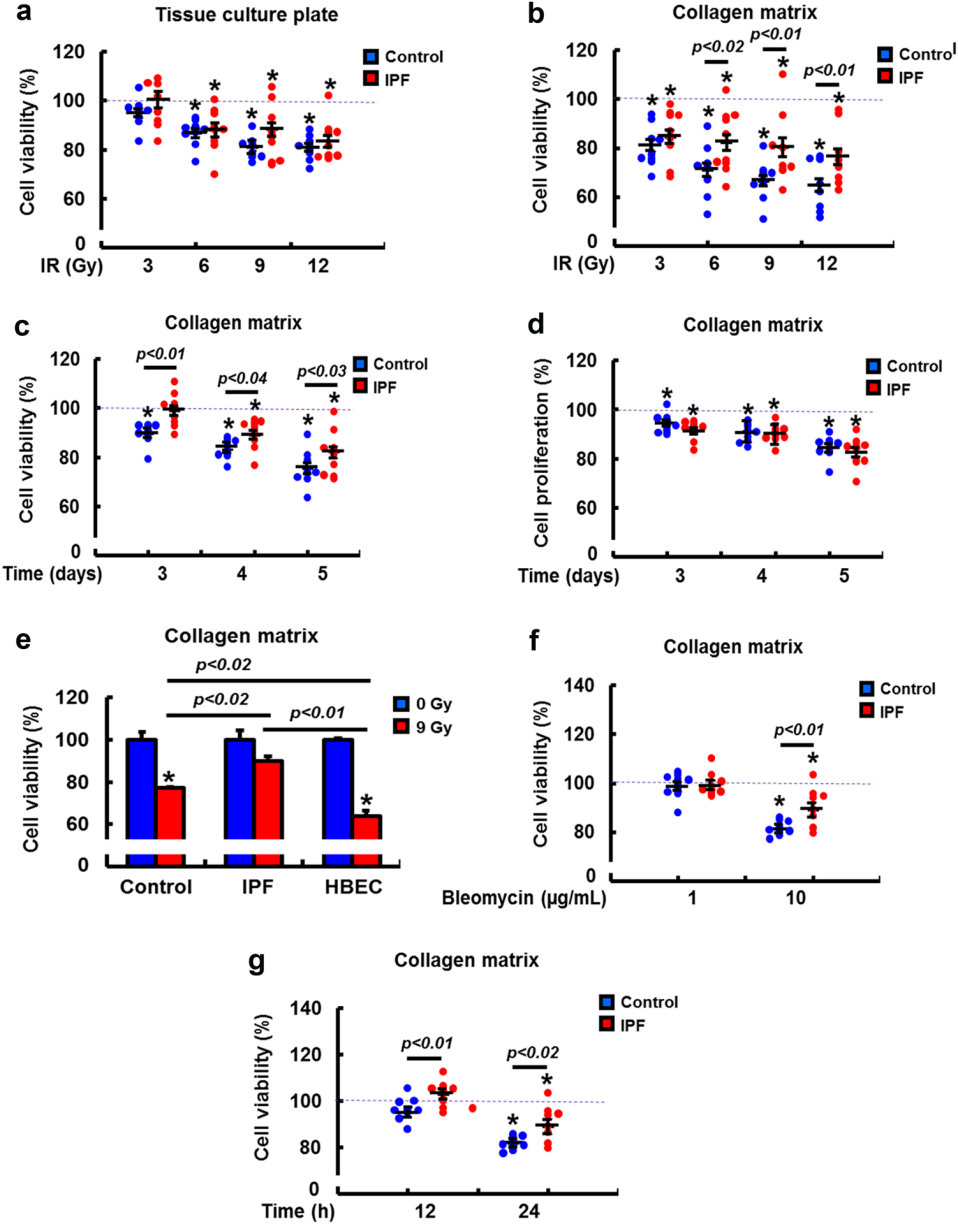
IPF fibroblasts are highly viable in response to genotoxic insults when cultured on collagen. **a** Cell viability of randomly selected control and IPF fibroblasts (*n* = 8, each) on a tissue culture plate at 5 days after 3–12 Gy radiation. **b** Cell viability of randomly selected control and IPF fibroblasts (*n* = 8, each) on a collagen matrix at 5 days after 3–12 Gy radiation. **c** Cell viability in control and IPF fibroblasts (*n* = 8, each) on collagen matrix as a function of time after 9 Gy radiation. **d** Cell proliferation in control and IPF fibroblasts (*n* = 8, each) on collagen as a function of time after 9 Gy radiation. **e** Comparison of cell viability among IPF fibroblasts, control fibroblasts, and HBEC3-KT (triplicates) cultured on collagen at 3 days after 9 Gy radiation. **f** Cell viability of control and IPF fibroblasts treated with various doses (1 and 10 µg/ml) of bleomycin on collagen for 24 h. **g** Cell viability of control and IPF fibroblasts treated with 10 µg/ml of bleomycin on collagen as a function of time. Two-way ANOVA was used to analyze viable control and IPF fibroblasts. Values are presented in mean ± SEM of percentages compared to unirradiated or non-bleomycin-treated control and IPF fibroblasts set at 100% (dotted line). *: statistical significance of cell viability compared to unirradiated or non-bleomycin-treated control, IPF, and HBEC3-KT cells at *p* < 0.05

As radiation is a risk factor for organ fibrosis, irradiated fibrotic fibroblasts likely also acquire pathological properties and become resistant to death. To test this, radiation dose-response viability was measured in IPF and control lung fibroblasts in the presence or absence of collagen matrix. There was no significant difference between IPF and control fibroblasts when cells were cultured on a tissue culture plate (Fig. 1a, Supplementary Figure S1a). However, IPF fibroblasts on collagen were significantly more viable after radiation compared to control fibroblasts (Fig. 1b, c, Supplementary Figure S1b and c). In contrast, there was no significant difference in cell proliferation between IPF and control fibroblasts following radiation (Fig. 1d, Supplementary Figure S1d). The failure of lung re-epithelization and the selection of highly viable fibrotic fibroblasts may contribute to the development of RILF, thus we examined the effects of radiation on bronchial epithelial cell viability. Epithelial cells were highly sensitive to radiation-induced death (Fig. 1e, Supplementary Figure S1e), suggesting that radiation may contribute to the fibrotic process by preferentially selecting for pathologic, radioresistant fibroblasts while normal epithelial and mesenchymal cells are killed. To complement this data, we further examined whether IPF fibroblasts become resistant to an alternative DNA DSB-inducing genotoxic insult. For this assay, control and IPF fibroblasts viability were assessed following bleomycin exposure. Similar to irradiated fibroblasts, bleomycin-treated IPF fibroblasts were more resistant to cell death compared to control fibroblasts (Fig. 1f, Supplementary Figure S1f). Time course assay also showed increased viability of IPF fibroblasts after bleomycin treatment (Fig. 1g). These results strongly suggest that IPF fibroblasts utilize a generally conserved pathological mechanism to protect them from DNA damage.

### IPF fibroblasts develop fewer DNA DSB following genotoxic insults and are resistant to cell death

**Fig. 2:**
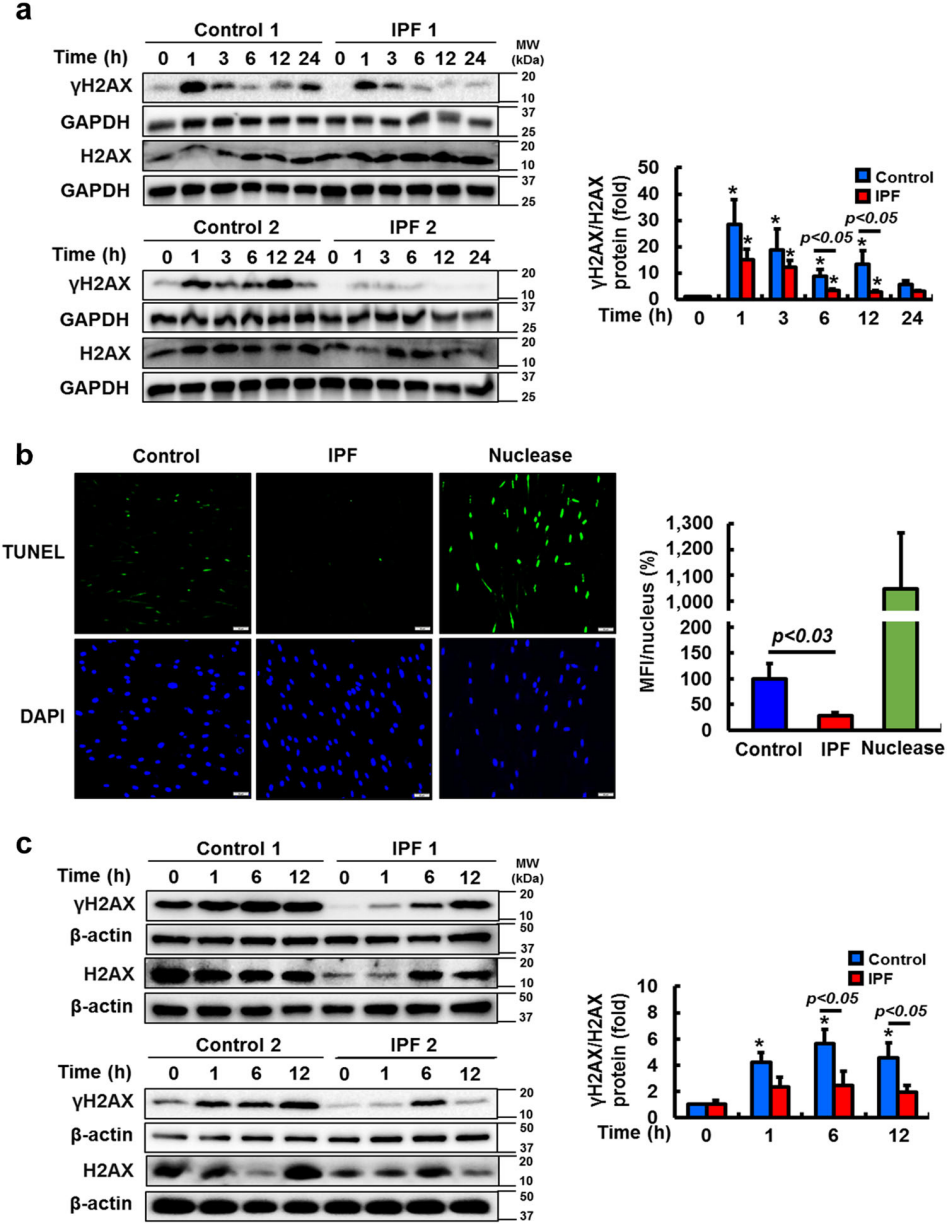
IPF fibroblasts show lower γH2AX expression and decreased DNA fragmentation following irradiation or bleomycin treatment. **a** Left, representative images showing γH2AX and total H2AX protein expression in randomly selected control and IPF fibroblasts (*n* = 8, each) as a function of time after 9 Gy radiation. GAPDH was used as a loading control. Right, statistical analysis of γH2AX/H2AX expression in control and IPF fibroblasts (*n* = 8, each) before and after radiation. Values are presented in mean ± SEM of fold changes compared to unirradiated control or IPF fibroblasts set at 1 fold. *: statistical significance of γH2AX/H2AX protein expression compared to unirradiated control or IPF fibroblasts at *p* < 0.05. **b** Left, representative images of DNA fragmentation in control and IPF fibroblasts (*n* = 4, each) on collagen at 24 h after 15 Gy radiation. Scale bar indicates 50 µm. Right, statistical analysis was obtained from the mean fluorescence intensity (MFI) per nucleus. Nuclease was used as a positive control. Values are presented in mean ± SEM of percentages compared to irradiated control fibroblasts set at 100%. **c** Left, representative images showing γH2AX and total H2AX protein expression in control and IPF fibroblasts (*n* = 8, each) as a function of time after bleomycin (10 µg/ml). Right, statistical analysis of γH2AX/H2AX expression in control and IPF fibroblasts (*n* = 8, each) as a function of time after bleomycin (10 µg/ml). Radioresistant IPF cells and radiosensitive control fibroblasts were selected for the experiment (as determined by viability following 9 Gy; Fig. 1b)

Immediately following a DNA DSB, large numbers of phosphorylated H2AX (γH2AX) molecules form around the break site to encourage accumulation of DNA repair proteins^33,34^. We measured γH2AX as an indirect indicator of DNA DSB, which correlates to radiation-induced cell death^33,35,36^. Radiation upregulated γH2AX expression in both IPF and control fibroblasts at 1 h (Fig. 2a, left), however predominantly resolved by 6 h in IPF cells. In contrast, sustained γH2AX expression was present in the majority of control lung fibroblasts. Reduced DNA DSB occurred in irradiated IPF cells compared to control fibroblasts, as demonstrated by reduced γH2AX/H2AX expression ratios (Fig. 2a, right). Moreover, TUNEL assay confirmed that IPF fibroblasts sustained less extensive DNA fragmentation and apoptosis compared to control fibroblasts (Fig. 2b). To further test if resistance of IPF fibroblasts to genotoxic stress is also due to reduced DNA DSB formation (Fig. 1f, g), we treated control and IPF fibroblasts with bleomycin, and γH2AX expression was measured. Reduced γH2AX levels were found in bleomycin-treated IPF fibroblasts compared to that of control fibroblasts as a function of time (Fig. 2c, left). Evaluation of γH2AX/H2AX expression ratios further demonstrated that DNA DSB remained low in most IPF fibroblasts (Fig. 2c, right). These findings support that IPF fibroblasts exert protective mechanisms to resist DNA DSB from both ionizing radiation and bleomycin, enabling this unique set of fibroblasts to maintain a resistant phenotype in response to genotoxic insults.

### Increased FoxM1 protects IPF fibroblasts from radiation-induced cell death by increasing RAD51 and BRCA2

**Fig. 3:**
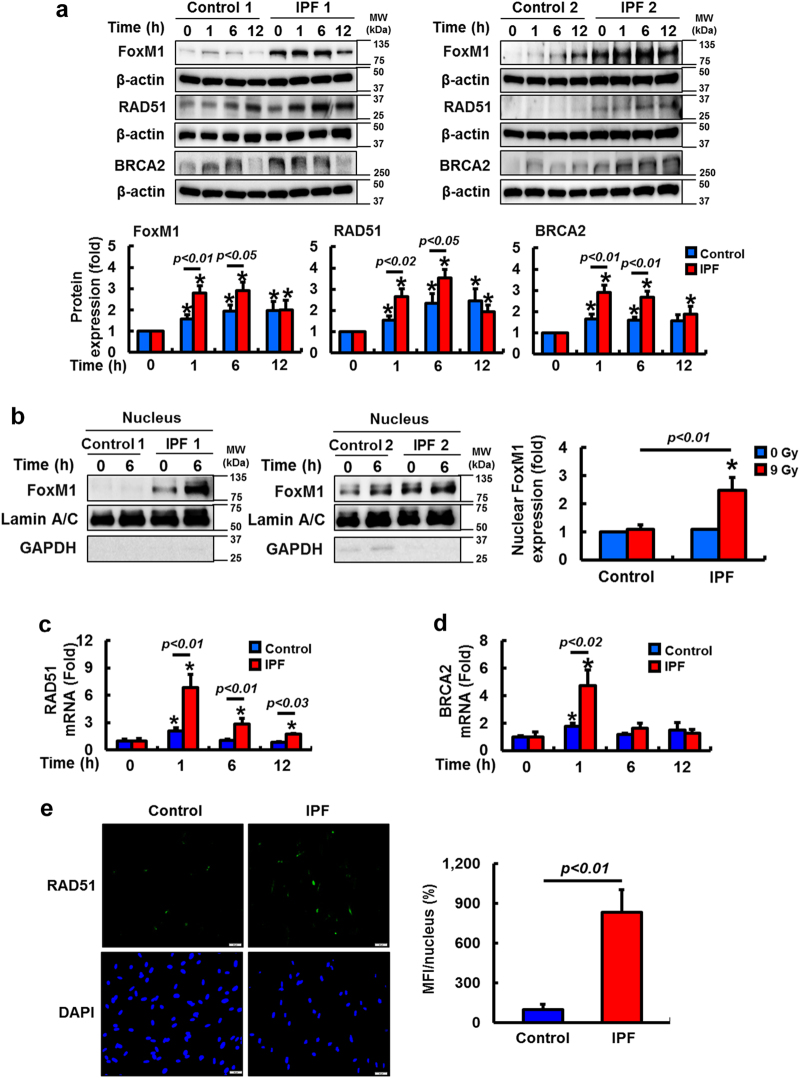
Radioresistant IPF fibroblasts upregulate FoxM1, RAD51, and BRCA2 following exposure to radiation. **a** Upper, representative images showing FoxM1, RAD51, and BRCA2 protein expression in randomly selected control (*n* = 8) and IPF (*n* = 8) fibroblasts before and after 9 Gy radiation. β-actin was used as a control. Lower, statistical analysis of FoxM1, RAD51, and BRCA2 protein expression in control and IPF fibroblasts (*n* = 8, each) before and after 9 Gy radiation. **b** Representative images and densitometry analysis of nuclear FoxM1 in control and IPF fibroblasts (*n* = 8, each) at 6 h after 9 Gy radiation. Lamin A/C and GAPDH were used as an internal control for nuclear and cytosolic fraction, respectively. **c** Changes in RAD51 mRNA expression in control and IPF fibroblasts (*n* = 8, each) as a function of time after 9 Gy radiation. **d** Changes in BRCA2 mRNA expression in control and IPF fibroblasts (*n* = 8) as a function of time after 9 Gy. Values are presented in mean ± SEM of fold changes compared to unirradiated control or IPF fibroblasts set at 1 fold. *: statistical significance of each protein or mRNA expression compared to unirradiated control or IPF fibroblasts at *p* < 0.05. **e** Left, control and IPF cells were irradiated with 9 Gy, and RAD51 positive cells were measured after 6 h as described in Materials and Methods. Right, statistical analysis was conducted using 3 images per each cell of 3 control or IPF fibroblasts. Scale bar indicates 50 µm. Shown is the mean fluorescence intensity (MFI) per nucleus. Values are presented in mean ± SEM of percentages compared to irradiated control fibroblasts set at 100%. Radioresistant IPF cells (high viability after 9 Gy) and radiosensitive control fibroblasts (low viability after 9 Gy) were selected for the experiment

FoxM1 dysregulation is implicated in the development of RILF^27–29^. FoxM1 regulates the expression of several DNA repair proteins including RAD51 and BRCA2, which are important for proper DNA DSB repair^27,28^. We therefore examined the role of FoxM1 on the expression of RAD51 and BRCA2 in irradiated IPF fibroblasts. Increased FoxM1 levels were found in IPF fibroblasts between 1 and 6 h compared to FoxM1 levels of irradiated control fibroblasts (Fig. 3a). Both RAD51 and BRCA2 were also high in the majority of IPF fibroblasts at similar time points to increased FoxM1 expression, further supporting that FoxM1 protects IPF fibroblasts from radiation-induced DNA damage via expression of RAD51 and BRCA2 (Fig. 3a, lower). We further examined whether FoxM1-dependent RAD51 and BRCA2 expression is altered in bleomyicn-treated IPF fibroblasts. Similar to the results observed in irradiated fibroblasts, FoxM1 was significantly increased in IPF fibroblasts compared to control fibroblasts (Supplementary Figure S2, upper and lower). Likewise, RAD51 and BRCA2 levels were increased in IPF fibroblasts compared to control fibroblasts after bleomycin treatment. These results support that a FoxM1-dependent increase in RAD51 and BRCA2 has a crucial role in protecting cells from genotoxic death via enhancing DNA repair. As a transcription factor, FoxM1 must translocate to the nucleus to activate its target genes^27,29^. Therefore, we next examined nuclear FoxM1 in IPF and control fibroblasts before and after radiation. Nuclear FoxM1 protein expression was predominantly increased in irradiated IPF fibroblasts compared to that of irradiated control fibroblasts (Fig. 3b). To test whether RAD51 and BRCA2 genes increase after radiation-induced FoxM1 upregulation, RAD51 and BRCA2 mRNA expression were measured over time (Fig. 3c, d). RAD51 and BRCA2 mRNA levels were increased at 1 h in irradiated IPF fibroblasts and remained high at 6 h compared to levels in control fibroblasts. Immunofluorescence further showed high nuclear RAD51 expression in irradiated IPF fibroblasts (Fig. 3e).

**Fig. 4:**
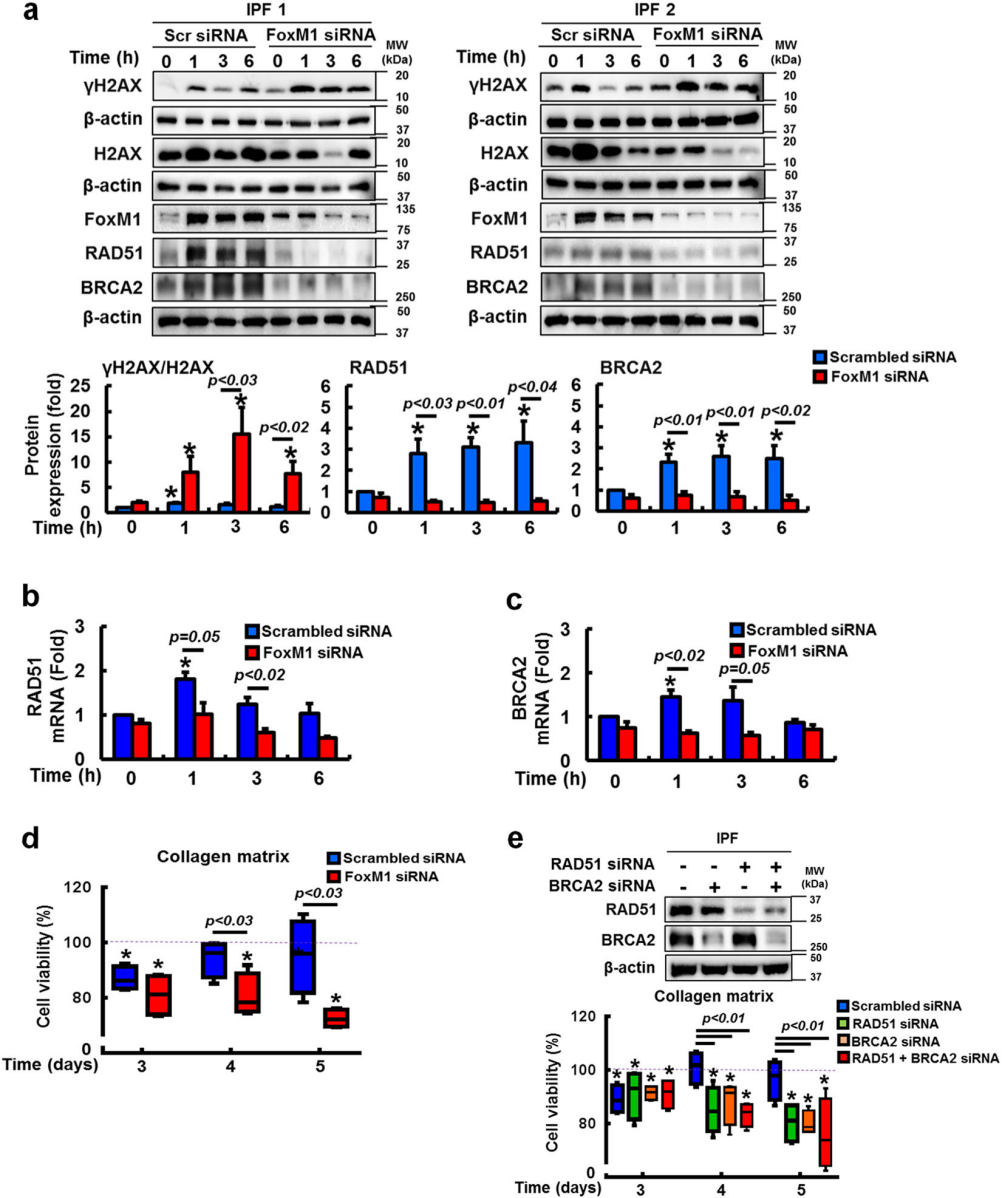
FoxM1 inhibition sensitizes IPF fibroblasts to radiation-induced cell death by suppressing RAD51 and BRCA2. **a** Upper, representative γH2AX, total H2AX, RAD51, and BRCA2 protein expression in FoxM1-silenced IPF fibroblasts (*n* = 4) before and after 9 Gy radiation. Lower, statistical analysis of γH2AX/H2AX, RAD51, and BRCA2 protein levels normalized to β-actin in IPF fibroblasts transfected with FoxM1 siRNA or scrambled (scr) siRNA as a function of time after 9 Gy radiation. **b** Effect of ionizing radiation on RAD51 mRNA levels in IPF fibroblasts (n = 3) that FoxM1 was silenced by siRNA. **c** Effect of ionizing radiation on BRCA2 mRNA levels in IPF fibroblasts (*n* = 3) that FoxM1 was silenced by siRNA. Scrambled siRNA was used as a control. Values are presented in mean ± SEM of fold changes compared to unirradiated IPF fibroblasts transfected with scrambled siRNA set at 1 fold. *: statistical significance of each protein or mRNA expression compared to unirradiated IPF fibroblasts transfected with scrambled siRNA at *p* < 0.05. **d** Changes in cell viability in FoxM1-silenced IPF fibroblasts (*n* = 4) following 9 Gy radiation. Values are presented in mean ± SEM of percentages compared to unirradiated IPF fibroblasts transfected with each siRNA set at 100% (dotted line). *: statistical significance of cell viability compared to unirradiated IPF fibroblasts transfected with each siRNA at *p* < 0.05. **e** Upper, RAD51 and BRCA2 silencing was confirmed by Western analysis. Lower, the effect of silencing RAD51, BRCA2, or both RAD51 and BRCA2 on IPF (*n* = 4) cell viability over time after 9 Gy. Values are presented in mean ± SEM of percentages compared to unirradiated IPF fibroblasts in each experimental group at 100% (dotted line). *: statistical significance of cell viability compared to unirradiated IPF fibroblasts in each experimental group at *p* < 0.05. IPF cells showing high cell viability in response to 9 Gy radiation were used for these experiments

**Fig. 5:**
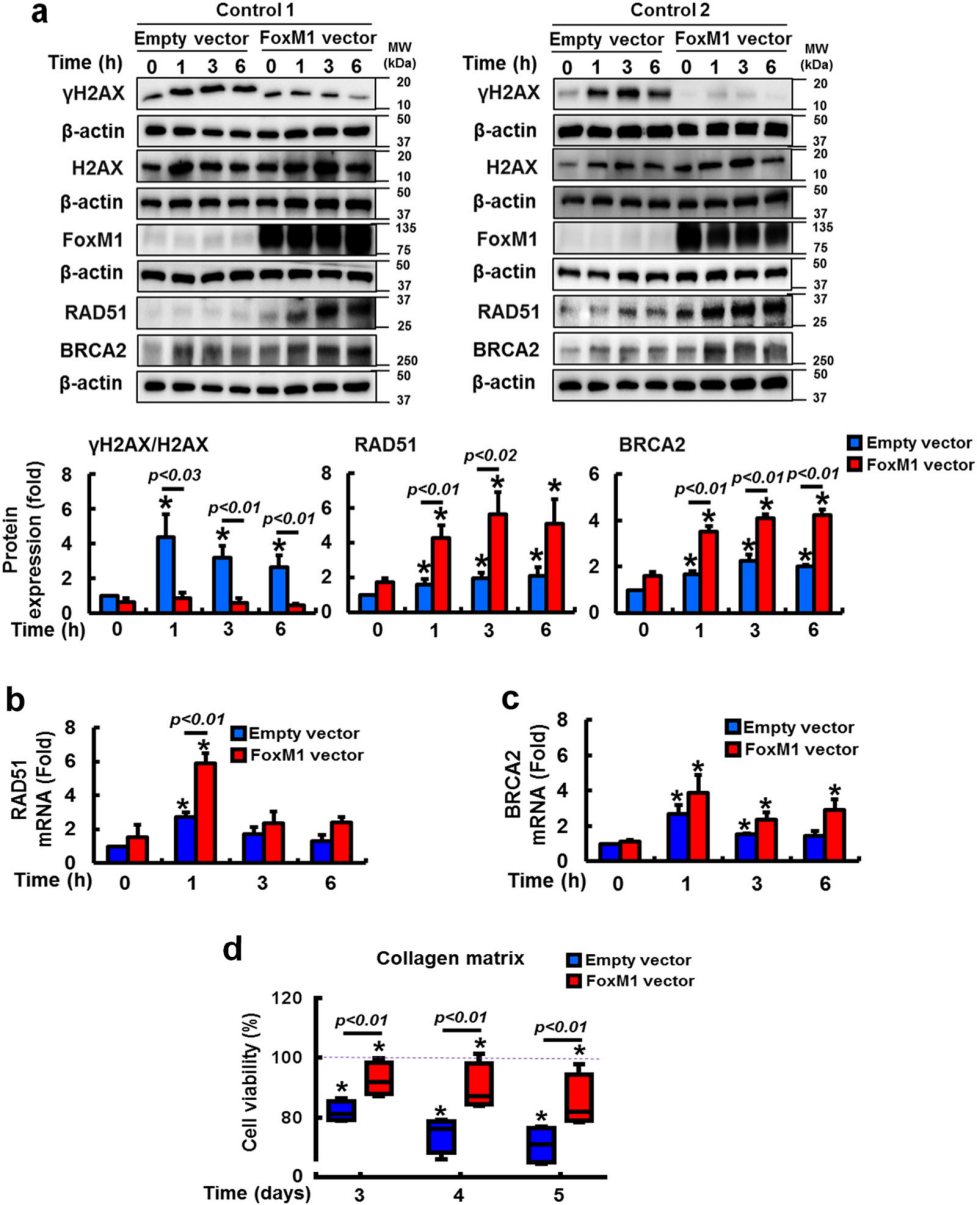
FoxM1 overexpression reduces DNA damage by upregulating RAD51 and BRCA2 in irradiated control fibroblasts. **a** Upper, effect of FoxM1 overexpression on γH2AX, total H2AX, RAD51, and BRCA2 protein expression in irradiated (9 Gy) control lung fibroblasts (*n* = 4). Lower, statistical analysis of γH2AX/H2AX, RAD51, and BRCA2 protein expression over time following 9 Gy radiation, normalized to β-actin. **b** The effect of radiation on RAD51 mRNA expression in control fibroblasts overexpressing FoxM1 or empty vector (*n* = 3, each). **c** The effect of radiation on BRCA2 mRNA expression in control fibroblasts overexpressing FoxM1 or empty vector (*n* = 3, each). Values are presented in mean ± SEM of fold changes compared to unirradiated control fibroblasts transfected with empty vector set at 1 fold. *: statistical significance of each protein or mRNA expression compared to unirradiated control fibroblasts transfected with empty vector at *p* < 0.05. **d** Cell viability in control fibroblasts (*n* = 4) overexpressing FoxM1 or empty vector following radiation at 9 Gy. Values are presented in mean ± SEM of percentages compared to unirradiated control fibroblasts transfected with each vector set at 100%. *: statistical significance of cell viability compared to unirradiated control fibroblasts transfected with each vector at *p* < 0.05. Radiosensitive control fibroblasts were selected for the experiment (cells with low viability following 9 Gy)

To confirm the direct role of FoxM1 on RAD51 and BRCA2 expression, we next examined γH2AX, RAD51 and BRCA2 protein levels in IPF fibroblasts in which FoxM1 was silenced. γH2AX expression was clearly increased in FoxM1-silenced IPF fibroblasts compared to that of scrambled siRNA-transfected cells (Fig. 4a, upper and lower). In contrast, RAD51 and BRCA2 protein expression were reduced in IPF fibroblasts when FoxM1 was silenced. To support the role of FoxM1 in regulating the transcriptional levels of RAD51 and BRCA2, FoxM1 silencing also significantly reduced gene expression of RAD51 and BRCA2 in irradiated IPF fibroblasts compared to control cells (Fig. 4b, c). Importantly, FoxM1-silenced fibroblasts became highly sensitized to radiation-induced cell death (Fig. 4d). To confirm the role of RAD51 and BRCA2, we next examined the impact of silencing RAD51 and BRCA2 on radiosensitivity in IPF fibroblasts. Inhibition of RAD51 and/or BRCA2 increased IPF cell death following radiation (Fig. 4e), supporting that altered RAD51–BRCA2 signaling contributes to radioresistance. To verify that FoxM1 regulates viability via RAD51 and BRCA2, we next examined the effect of radiation on control fibroblasts in which FoxM1 was overexpressed. γH2AX expression was decreased following radiation, while RAD51 and BRCA2 protein levels were increased (Fig. 5a). RAD51 and BRCA2 mRNA levels were also highly increased following FoxM1 overexpression (Fig. 5b, c). Furthermore, FoxM1 overexpression increased radioresistance compared to fibroblasts without enhanced FoxM1 activity (Fig. 5d). To further elucidate if alteration of FoxM1 affects the cellular phenotype via modulating RAD51 and BRCA2 in response to radiation, we next selected control and IPF fibroblasts that are resistant and sensitive to radiation-induced cell death, respectively (Fig. 1b). If the FoxM1/RAD51–BRCA2 axis is crucial in altering cellular responses against ionizing radiation, we hypothesized that cells would also express abnormal levels of FoxM1 and target DNA repair proteins, thereby changing a phenotype. Indeed, γH2AX expression was reduced in highly viable control fibroblasts (Con4) compared to radiosensitive control fibroblasts (Con1) (Supplementary Figure S3). FoxM1 and RAD51 expression profoundly increased in radioresistant control fibroblasts compared to that of radiosensitive control fibroblasts. Likewise, compared to radioresistant IPF fibroblasts (IPF4), increased γH2AX levels were found in radiosensitive IPF fibroblasts (IPF1). Finally, FoxM1, RAD51, and BRCA2 expression was highly increased in radioresistant IPF fibroblasts while the expression levels of these proteins remained low or relatively unaltered in radiosensitive IPF fibroblasts. Taken together, these results show that FoxM1 deregulation has an important role in protecting fibroblasts from radiation-induced cell death via enhanced RAD51 and BRCA2 repair activity.

### FoxO3a inhibition increases FoxM1-dependent DNA repair in irradiated IPF fibroblasts

**Fig. 6:**
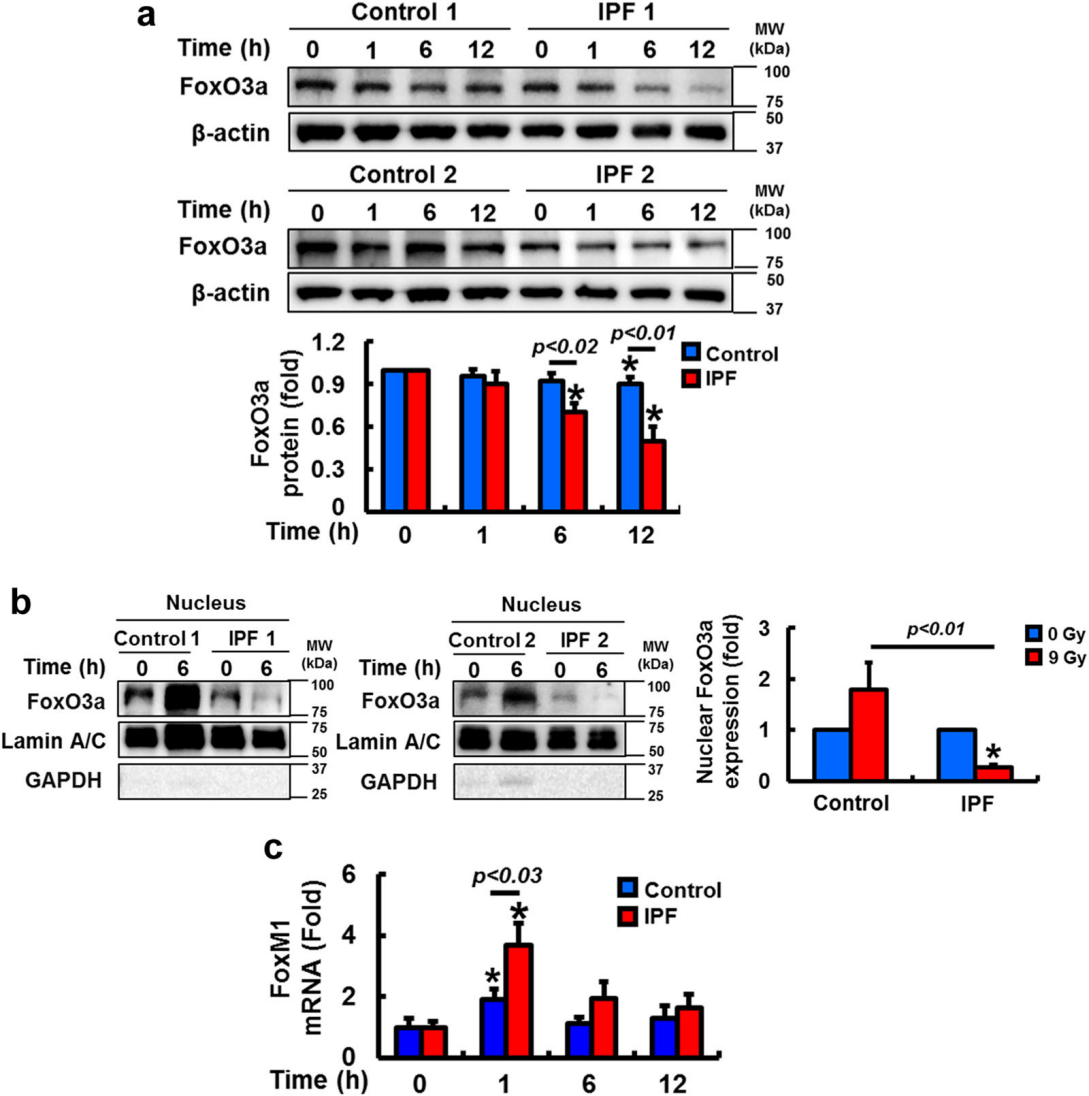
FoxO3a protein is reduced in IPF fibroblasts after radiation. **a** Upper, representative FoxO3a protein expression in control and IPF fibroblasts (n = 8, each) before and after 9 Gy radiation. Lower, Statistical analysis of FoxO3a expression in control and IPF fibroblasts (*n* = 8, each) normalized to β-actin under the same conditions. **b** Representative image of nuclear FoxO3a in control and IPF fibroblasts (*n* = 8, each) at 6 h after 9 Gy. Lamin A/C and GAPDH were used as a nuclear and cytosolic internal control, respectively. **c** FoxM1 mRNA levels in control and IPF fibroblasts (*n* = 8, each) as a function of time after radiation (9 Gy). Values are presented in mean ± SEM of fold changes compared to unirradiated control or IPF fibroblasts set at 1 fold. *: statistical significance of each protein or mRNA expression compared to unirradiated control or IPF fibroblasts at *p* < 0.05

**Fig. 7:**
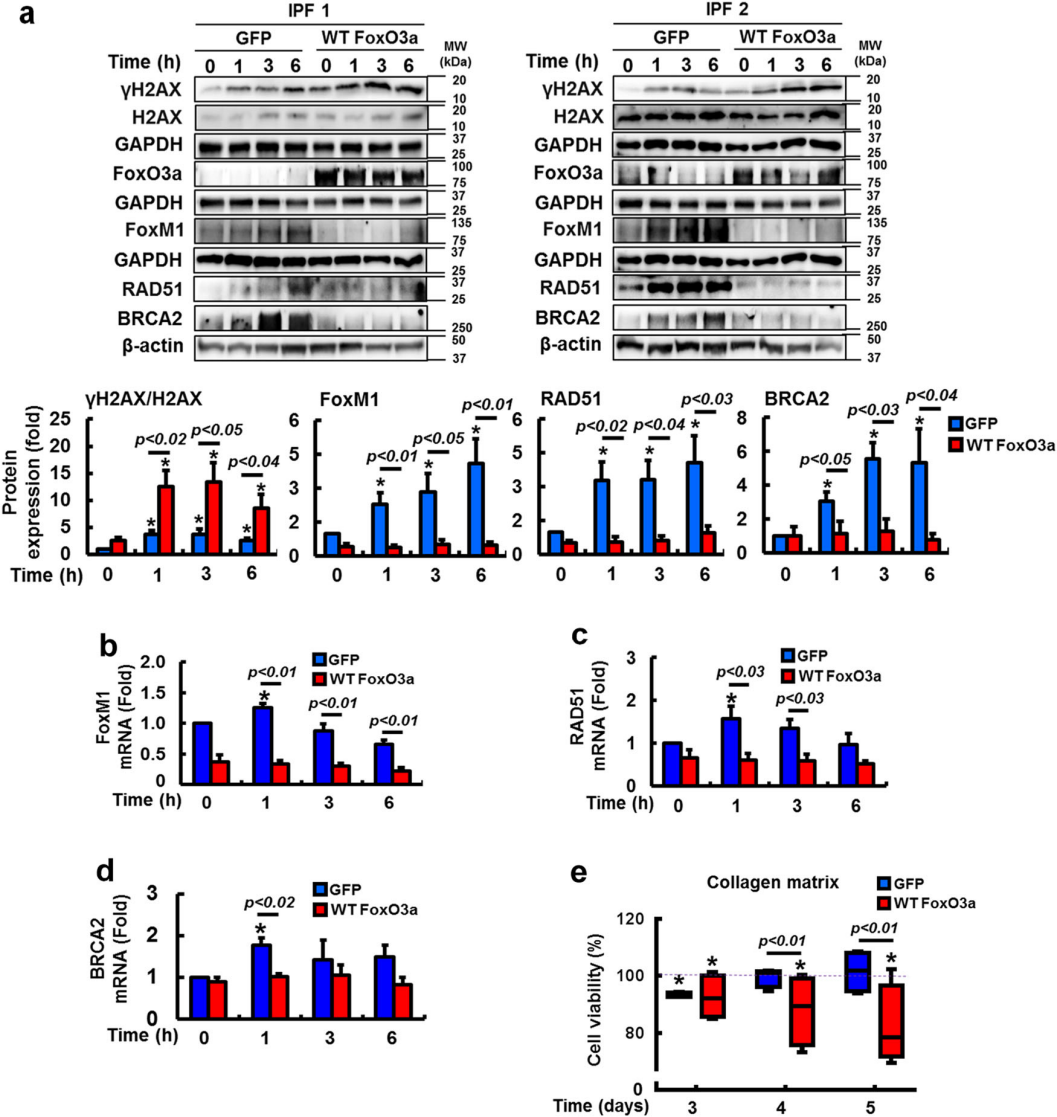
FoxO3a reconstitution increases DNA damage and decreases DNA repair protein expression. **a** Upper, representative images of γH2AX, total H2AX, FoxM1, RAD51, and BRCA2 protein expression in IPF fibroblasts overexpressing wildtype FoxO3a (WT FoxO3a; *n* = 4). GFP: as a control. Lower, statistical analysis of γH2AX/H2AX, FoxM1, RAD51, and BRCA2 protein expression normalized to β-actin or GAPDH over time following 9 Gy radiation. Effect of FoxO3a overexpression in IPF fibroblasts on **b** FoxM1, **c** RAD51, and **d** BRCA2 mRNA expression after radiation compared to IPF fibroblasts expressing GFP (*n* = 3). Values are presented in mean ± SEM of fold changes compared to unirradiated IPF fibroblasts overexpressing GFP set at 1 fold. *: statistical significance of each protein or mRNA expression compared to unirradiated IPF fibroblasts overexpressing GFP at *p* < 0.05. **e** Changes in cell viability in IPF fibroblasts (*n* = 4) overexpressing wildtype FoxO3a after radiation at 9 Gy. IPF fibroblasts overexpressing GFP was used as a control. Values are presented in mean ± SEM of percentages compared to unirradiated IPF fibroblasts overexpressing GFP or WT FoxO3a set at 100% (dotted line). *: statistical significance of cell viability compared to unirradiated IPF fibroblasts overexpressing GFP or WT FoxO3a at *p* < 0.05. Radioresistant IPF fibroblasts were selected for the experiment (cells with high viability following 9 Gy)

**Fig. 8:**
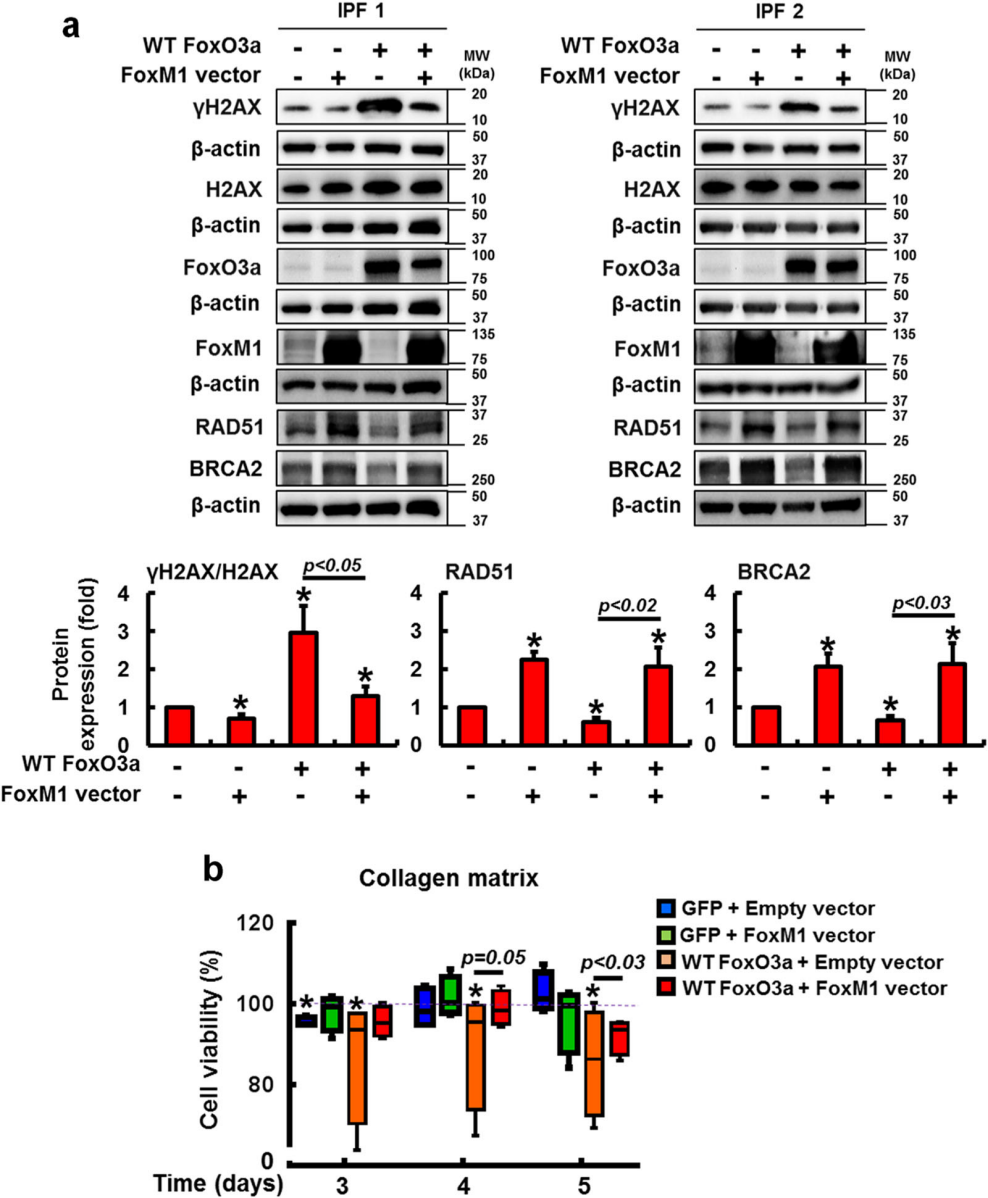
FoxO3a regulates RAD51–BRCA2-mediated DNA repair pathway via FoxM1. **a** Upper, representative images of γH2AX, total H2AX, RAD51, and BRCA2 protein expression in IPF fibroblasts overexpressing FoxO3a or FoxM1, or FoxO3a together with FoxM1 at 6 h after 9 Gy radiation. Lower, statistical analysis of γH2AX/H2AX, RAD51, and BRCA2 protein expression in IPF fibroblasts (*n* = 4, each) under the same conditions. Values are presented in mean ± SEM of fold changes compared to irradiated IPF fibroblasts overexpressing GFP and empty vector set at 1 fold. *: statistical significance of each protein compared to irradiated IPF fibroblasts overexpressing GFP and empty vector at *p* < 0.05. **b** Cell viability of IPF fibroblasts (*n* = 4, each) under the same condition as described above. Values are presented in mean ± SEM of percentages compared to unirradiated IPF fibroblasts in each experimental group at 100%. *: statistical significance of cell viability compared to unirradiated IPF fibroblasts in each experimental group at *p* < 0.05. Radioresistant IPF fibroblasts (high viability after 9 Gy) were selected for these experiments

FoxO3a is abnormally suppressed in IPF fibroblasts and confers protection from collagen matrix-driven apoptosis^8–11,31,32^. Recent studies suggested that FoxO3a transcriptionally suppresses FoxM1 and that an inverse correlation between FoxO3a and FoxM1 expression exists^27,30^. To test this possibility, we examined FoxO3a expression in irradiated IPF and control fibroblasts. FoxO3a levels predominantly decreased in IPF fibroblasts compared to that of control fibroblasts as a function of time (Fig. 6a). Moreover, nuclear FoxO3a remained low in IPF fibroblasts while enhanced FoxO3a was found in control fibroblasts following radiation (Fig. 6b). Additionally, enhanced FoxM1 mRNA expression was found in IPF fibroblasts compared to that of control fibroblasts after radiation (Fig. 6c). These results suggest that FoxO3a suppression in irradiated IPF fibroblasts participates in the transcriptional upregulation of FoxM1. Since FoxO3a is abnormally low in IPF fibroblasts on collagen, we next reconstituted FoxO3a in IPF fibroblasts, and FoxM1, RAD51, and BRCA2 protein levels were measured on collagen following radiation. IPF fibroblasts overexpressing FoxO3a showed high γH2AX expression while protein levels of the FoxM1 and its target RAD51 and BRCA2 were reduced (Fig. 7a), suggesting that FoxO3a regulates a FoxM1-dependent DNA repair pathway. To further support that FoxO3a modulates this pathway, FoxM1, RAD51, and BRCA2 mRNA expression levels were also significantly reduced following radiation in FoxO3a overexpressing IPF fibroblasts compared to IPF fibroblasts overexpressing GFP (Fig. 7b–d). FoxO3a overexpression also significantly sensitized IPF fibroblasts to radiation-induced cell death as a function of time (Fig. 7e). Collectively, our results suggest that abnormally low FoxO3a activity in IPF fibroblasts confers radioresistance through FoxM1 activation, which subsequently upregulates RAD51 and BRCA2 repair activity. To confirm that FoxM1/RAD51–BRCA2 pathway activity is FoxO3a-dependent in irradiated fibroblasts, we next examined the effect of radiation on the protein expression of γH2AX, RAD51, BRCA2, and the viability of IPF fibroblasts co-overexpressing FoxO3a and FoxM1. When FoxM1 alone was overexpressed, γH2AX expression was decreased while RAD51 and BRCA2 expression levels were increased (Fig. 8a). In contrast, when FoxO3a alone was overexpressed, γH2AX expression was increased while RAD51 and BRCA2 expression was reduced. However, when FoxM1 was reconstituted in IPF fibroblasts overexpressing FoxO3a, γH2AX expression was decreased compared to FoxO3a overexpression alone, with RAD51 and BRCA2 expression restored to levels similar to those following FoxM1 overexpression alone. Moreover, FoxO3a sensitized IPF fibroblasts to radiation-induced cell death while reconstitution of FoxM1 in FoxO3a overexpressing IPF fibroblasts provided radioprotection (Fig. 8b). Collectively, our results demonstrate that decreased radiosensitivity in IPF fibroblasts occurs through a FoxO3a-dependent FoxM1/RAD51–BRCA2 survival pathway.

## Discussion

The presence of persistent fibrotic fibroblasts is closely associated with the progression of IPF, thus it is crucial to elucidate how these fibroblasts maintain their aberrantly viable phenotype in response to unfavorable conditions. We therefore sought to examine whether fibroblasts derived from IPF patients are also resistant to radiation; not only does this study provide insight into the pathological IPF fibroblast phenotype but it may shed light on the development of RILF. We found that lung fibroblasts from patients without IPF are more radiosensitive than IPF fibroblasts. Contrary to IPF fibroblasts, bronchiolar and alveolar epithelial cells are more sensitive to radiation injury, even at relatively low doses of radiation^37,38^. Our data also support that radiation preferentially damages normal lung epithelial cells and fibroblasts, whereas resident pathological pro-fibrotic fibroblasts, which may be inhabitant in small numbers, are likely to survive and contribute to the fibrotic process. Fibrosis-inducing cells such as IPF fibroblasts flourish in collagen-rich conditions^9,10,32^, therefore the normal healing process following radiation injury may provide an environment that favors fibrosis. It is feasible that the presence of even a small population of pro-fibrotic lung fibroblasts in cancer patients may be at an increased risk of RILF following treatment. Currently, it is thought that IPF develops following aberrant lung epithelial cell death as a result of chronic, but often unidentified, lung injury^39–43^. Fibroblasts become activated by various cytokines and growth factors, which contribute to the development of IPF. Patients with early-stage IPF are challenging to diagnose and most patients present with advanced-disease; older patients with cancer may harbor IPF fibroblasts that predispose to progressive fibrosis after radiation treatment. It is therefore important to detect fibrotic fibroblasts such as IPF fibroblasts in cancer patients who will sustain radiation dose to the lungs to better predict the likelihood of clinically relevant RILF. Efforts to identify and selectively promote death of these radioresistant fibroblasts may also lead to a more appropriate healing process within irradiated lung.

Our prior studies demonstrated that abnormally low FoxO3a protects IPF fibroblasts from collagen-rich matrix-driven apoptosis^9,10,32^. In this study, we elucidated that FoxO3a alteration directly contributes to reduced DNA damage via the FoxM1/RAD51–BRCA2-dependent axis, fortifying the crucial role of FoxO3a in IPF fibroblasts. Our data further demonstrates that radiosensitivity in IPF cells can be altered through manipulation of the FoxO3a–FoxM1 axis. Therapeutic attempts to promote the cell death of fibrotic fibroblasts may be fruitful if FoxM1 activity is suppressed by normal FoxO3a function. Our study here may be important when addressing the pathological role of fibroblasts in other diseases, such as the role that cancer-associated fibroblasts have in promoting tumor growth and affecting prognosis^32,44,45^. FoxO3a–FoxM1 dysregulation is implicated in carcinogenesis, tumor progression, and therapeutic resistance, highlighting the need to understand their relationship and downstream effectors^46–49^. FoxM1 alone is a major predictor of adverse outcome in several carcinomas, including carcinoma of the breast, ovary, lung, liver, and stomach^47–50^. Likewise, we found that IPF fibroblasts utilize FoxM1 to protect them from radiation-induced DNA damage and subsequent cell death, suggesting that FoxM1 activity is important in promoting cell survival. Our study is unique in that eight non-immortalized cell lines were derived directly from patients with IPF, thus providing a rare opportunity to evaluate for inherent biologic variability. This strengthens our findings as we have utilized a wide variety of mortal cell lines for both the IPF and non-IPF fibroblast cell lines and discovered significant differences between groups. There is no reliable method of predicting the development of RILF and current efforts aim to reduce the dose of radiation to lung, which potentially lessens tumor control. As it is difficult to detect resident IPF fibroblasts in cancer patients, FoxM1 expression may be a hallmark of abnormal fibroblast phenotype and serve as a prognostic marker indicative of a higher risk of RILF. FoxM1 may therefore be a viable target to prevent the development and progression of IPF, RILF, and other fibrotic diseases.

In summary, our data demonstrate that pathologic IPF fibroblasts are radioresistant with enhanced survival following via enhanced DNA repair. We propose that early detection and selective targeting of FoxM1-expressing fibrotic fibroblasts will mitigate the adverse events associated with lung irradiation and improve long-term outcome in cancer survivors. This approach may be broadly applicable to other fibrotic diseases.

## Materials and methods

### Human subjects and the isolation of primary lung fibroblasts

Lung tissues removed at the time of transplantation or death from non-IPF and IPF patients (*n* = 8, each). The tissue samples were stripped of all identifiers and designated as waste (exemption 4). Written informed consent was obtained on all patients prior to the procedure being performed. Use of human lung tissues was approved by the Institutional Review Board (IRB) at the University of Minnesota. The diagnosis of IPF was supported by history, physical examination, pulmonary function tests, and typical high-resolution chest computed tomographic findings of IPF. In all cases, the diagnosis of IPF was confirmed by microscopic analysis of lung tissue that demonstrated the characteristic morphological findings of interstitial pneumonia^51,52^. Removed lung tissues were chopped to 5 mm^3^ and cultured in Dulbecco’s modified Eagle’s medium (DMEM; MilliporeSigma, St. Louis, MO, USA) supplemented with 20% fetal calf serum (FCS; HyClone, Logan, UT, USA) and 2% antibiotics for 4–5 weeks at 37 °C in a 5% CO_2_ humidified incubator to prepare IPF and non-IPF (control) lung fibroblasts. They were used at passage numbers 3–9 for all experiments.

### Collagen matrix and epithelial cells

Collagen matrix was prepared using 80% of type I collagen solution (Advanced Biomatrix, San Diego, CA, USA), 10% 10× DMEM (MilliporeSigma) and 10% 1×DMEM, and the pH was adjusted to 7.2 with 0.1 M NaOH. Cell culture dishes or 96-well plates were coated with collagen and incubated for a minimum of 2 h at 37 °C prior to use. Following 24 h, serum-free DMEM (SF DMEM) was used for experiments. Human Bronchial Epithelial Cell3-KT (HBEC3-KT) cell line was obtained from American Type Culture Collection (ATCC, Manassas, VA, USA) and cultured in airway epithelial cell basal medium (ATCC) supplemented with 1% antibiotics and bronchial epithelial cell growth kit (ATCC) at 37 °C.

### Ionizing radiation

Radiation was delivered using 6 MV photons from a clinical linear accelerator (Varian Clinac IX; Varian Medical Systems, Palo Alto, CA, USA) routinely calibrated and maintained to clinical standards. Cells were treated with increasing radiation doses (0–15 Gy). Culture plates were placed on 1.5 cm of tissue equivalent material and the gantry rotated to 180 degrees to ensure electronic equilibrium at the level of cells. Radiation was prescribed using a single source-to-surface distance technique.

### Cell viability and proliferation assays

Randomly selected non-IPF control lung fibroblasts (*n* = 8) and IPF fibroblasts (*n* = 8) were cultured on collagen-coated or non-collagen-coated 96-well plates in SF DMEM prior to radiation or bleomycin (Alfa Aesar, Ward Hill, MA, USA). Cells were then incubated for 3 h with 20 μL CellTiter Blue reagent (Promega, Madison, WI, USA) to assess viability or with 20 μL CellTiter 96 Aqueous One Solution (Promega) to assess proliferation for 3 h. Cell viability and proliferation were measured at 560 nm (excitation)/590 nm (emission) of fluorescence and at 490 nm of absorbance using a 96-well plate reader (BioTek, Winooski, VT, USA), respectively.

### TUNEL assay

Deoxynucleotidyltransferase (TdT)-mediated dUTP nick-end labeling (TUNEL) assay was conducted using In Situ Cell Death Detection kit (Roche Applied Science, Indianapolis, IN) according to manufacturer’s instruction. Briefly, 4 of each control and IPF fibroblasts plated on collagen matrix coated cover slips (ThermoFisher Scientific, Pittsburgh, PA, USA) in SF DMEM were irradiated with 15 Gy and cells were then incubated for an additional 24 h. Control cells showing low cell viability and IPF cells showing high cell viability in response to the radiation were chosen for the current assay. After the incubation, cells on cover slips were washed with phosphate buffered saline (PBS; pH 7.4) and fixed with 4% paraformaldehyde solution (USB, Cleveland, OH, USA) at 4 °C over night. Cells on cover slips were then permeabilized with Proteinase K (DAKO, Carpinteria, CA, USA) at room temperature for 20 min. After the washing with PBS, cells were incubated with TUNEL reaction mixture at 37 °C for 2 h and washed with PBS twice. In addition, the fixed control cells on cover slips was incubated with Nuclease (Trevigen, Gaithersburg, MD, USA) at 37 °C for 25 min before incubating in TUNEL reaction mixture to be used as a positive control. For fluorescence microscopic analysis, the cover slips were washed with PBS and sequentially moved on slide glasses having mounting solution with DAPI (Life Technologies, Carlsbad, CA, USA). The images were taken from two different areas on each cover slip using a confocal fluorescence microscopy (Axio plan2, Zeiss, Oberkochen, Germany) at ×200 magnification, and the images were processed using Progres Capture Pro V2.8.8 software (Jenoptik, Jena, Germany). Images analysis was conducted using ImageJ program (NIH, Rockville, MD).

### Western blot analysis

Same control and IPF fibroblasts (*n* = 8, each) used in cell viability assay (6 × 10^5^ cells/60 mm cell culture dish) were irradiated with  9 Gy and incubated for the assigned time periods. After the incubation, cells were lysed with 1×cell lysis buffer (Cell Signaling technology, Beverly, MA, USA) containing protease inhibitor (Roche Applied Science) and phosphatase inhibitor (Research Products International, Mount Prospect, IL, USA). Cell lysates were collected, sonicated on ice for 15 s, and then denatured using 5×Laemmli buffer at 95℃ for 10 min. The lysates were separated by a gradient polyacrylamide gels having 5–20% polyacrylamide concentration and proteins on the gel were electrically transferred to a PVDF membrane (Bio-Rad, Hercules, CA, USA) using a Protean III tank transfer system (Bio-Rad). After blocking step using 5% skim milk in TTBS (0.1 M Tris, 0.9% NaCl, and 0.1% Tween 20) for 1 h at room temperature, the membrane was incubated with γH2AX (catalog No. 9718, Cell Signaling technology), H2AX (catalog No. 2595, Cell Signaling technology), FoxM1 (catalog No. SC-376471, Santa Cruz Biotechnology, Santa Cruz, CA, USA), FoxO3a (catalog No. 07-1719, Millipore, Bedford, MA, USA), RAD51 (catalog No. ab88572, Abcam, Cambridge, UK), BRCA2 (catalog No. MAB2476, R&D systems, Minneaplis, MN, USA), GAPDH (catalog No. SC-32233, Santa Cruz Biotechnology), Lamin A/C (catalog No. 2032, Cell Signaling technology) or β-actin (catalog No. 4967, Cell Signaling technology) antibody diluted in TTBS containing 5% bovine serum albumin (BSA) at 4℃ for 24–48 h. After washing with TTBS three times, membranes were incubated with anti-rabbit or mouse IgG antibody conjugated with horseradish peroxidase (MilliporeSigma) diluted in TTBS containing 5% skim milk for 1 h at room temperature. The protein bands on a membrane were detected by ECL solution (ThermoFisher Scientific) using Chemi Doc-IT2 image analyzer (UVP BioImage systems, Upland, CA, USA), and quantified using VisionWorks LS program (UVP BioImage systems).

### Nuclear protein isolation

Nuclear protein fractions were isolated from control (*n* = 8) and IPF (*n* = 8) fibroblasts using NE-PER Nuclear and Cytoplasmic Extraction Reagents kit (ThermoFisher Scientific) according to the manufacturer’s instructions. Briefly, control and IPF fibroblasts (1.6 × 10^6^ cells/10 cm cell culture dish) cultured on collagen in SF DMEM were incubated for 6 h after 9 Gy radiation. Cells were lysed with Cytoplasmic Extraction Reagent I and II. Cytosolic fraction was collected by centrifugation at 16,000×*g* for 5 min and the remaining pellet was lysed with Nuclear Extraction Reagent. Nuclear fraction was then isolated by  centrifugation at 16,000×*g* for 10 min. Both cytosolic and nuclear fractions were denatured with 5×Laemmli buffer at 95 °C for 10 min, and FoxM1 and FoxO3a protein expression was measured using Western analysis. Lamin A/C and GAPDH were used as a nuclear and cytosolic protein marker, respectively.

### Real-time polymerase chain reaction (PCR)

Total RNA from control or IPF fibroblasts (*n* = 8, each) cultured (3 × 10^5^ cells/35 mm cell culture dish) on collagen was extracted at specified time points following 9 Gy radiation using TRIGent (Biomatik, Cambridge, ON, Canada). One µg of total RNA was synthesized into complementary DNA, and PCR was conducted using Step One Plus real-time PCR system (Applied Biosystems, Foster city, CA, USA) and Power SYBR Green PCR master mix (Applied Biosystems) under the following conditions: initial denaturation at 95 °C for 10 min then amplification by cycling 45 times at 95 °C for 15 s, 55 °C for 30 s, then 68 °C for 45 s. GAPDH expression was examined to normalize the copy numbers of each gene. Primer Sequences for PCR: FoxO3a (Forward: 5′-AAATGTTCGTCGCGGCGGAAC-3′; Reverse: 5′-GTCGCCCTTATCCTTGAAGTA-3′), FoxM1 (Forward: 5′-AAGCCAGGCTGGAAGAACTC-3′; Reverse: 5′-ATGTCAAGTAGCGGTTGGCA-3′), RAD51 (Forward: 5′-TTGGCCCACAACCCATTTCA-3′; Reverse: 5′-TTAGCTCCTTCTTTGGCGCA-3′), BRCA2 (Forward: 5′-ACAAAGGCAACGCGTCTTTC-3′; Reverse: 5′-TGAGAACACGCAGAGGGAAC-3′) and GAPDH (Forward: 5′-TTCATTGACCTCAACTACATGGT-3′; Reverse: 5′-CCTTCTCCATGGTGGTGAAGA-3′).

### siRNA, adenovirus, and vector

IPF fibroblasts (*n* = 4) were transfected with FoxM1, BRCA2, RAD51, or control siRNA (catalog No. SC-43769, SC-29825, SC-36361, SC-37007, Santa Cruz Biotechnology) using Lipofectamin RNAiMAX (Life technologies). For overexpression of FoxM1, control fibroblasts (*n* = 4) were transfected with 2 µg empty pCMV3 or FoxM1-pCMV3 vector (Sino Biological, Beijing, China) using Lipofectamin 3000 (Life technologies) in Opti-MEM (Gibco, Carlsbad, CA, USA). For FoxO3a overexpression, IPF fibroblasts (*n* = 4) were infected with either 2 × 10^6^ PFU of ad-GFP-FOXO3a (catalog No. NC09602131, Vector Biolabs, Philadelphia, PA, USA) or Ad-GFP (catalog No. 1060, Vector Biolabs) according to the manufacturer’s instructions. For co-overexpression experiments, IPF fibroblasts (*n* = 4) infected with ad-GFP-FOXO3a were transfected with FoxM1-pCMV3 vector as described above. Cells were then cultured on collagen in SF DMEM for 24 h followed by irradiation (9 Gy). FoxM1 overexpression or silencing by siRNA was verified by measuring FoxM1 mRNA (Supplementary Figure S4a and b).

### Immunofluorescence staining

Control and IPF fibroblasts (*n* = 3, each) cultured on collagen in SF DMEM were irradiated at 9 Gy followed by incubation for an additional 6 h at 37 °C. Cells were then fixed with 4.0% paraformaldehyde in PBS and permeabilized in 0.1% Triton-X (MilliporeSigma) in PBS for 15 min. The cells were incubated with the anti-RAD51 antibody (catalog No. ABE257, MilliporeSigma) diluted 1:300 in PBS at 4 °C for 24 h. Cells were then incubated with an anti-rabbit Alexa Fluor 488 conjugated secondary antibody (catalog No. A11008, ThermoFisher Scientific) diluted 1:250 in PBS for 20 min. Cells on collagen matrix were cover slipped with ProLong Gold Antifade Mountant with DAPI (ThermoFisher Scientific). From each sample, 3 of ×20 magnified images were analyzed using an upright microscope (Olympus BX53 microscope, Olympus DP73 camera; Olympus America, Center Valley, PA, USA). Images analysis was conducted using ImageJ (NIH).

### Statistics

Data are expressed as the means ± SEM. Box-and-whisker and dot plots were created with Prism V7.0 (GraphPad Software, La Jolla, CA, USA), showing lowest, lower quartile, median, upper quartile and highest expression levels. Two-dimensional column graphs were prepared with Microsoft Excel. Comparison between unirradiated and irradiated fibroblasts or comparison between irradiated IPF and control fibroblasts as a function of time was conducted using two-tailed Student’s *t*-test for Western analysis or two-way ANOVA for cell viability and proliferation assays by Prism V7.0. Significance level was set at *p* < 0.05.

## Electronic supplementary material

Supplementary Figure 1

Supplementary Figure 2

Supplementary Figure 3

Supplementary Figure 4

Supplementary Figure 5

Supplementary figure legends
