# Supplementary figures and images for: FoxM1-dependent RAD51 and BRCA2 signaling protects idiopathic pulmonary fibrosis fibroblasts from radiation-induced cell death
Source: Cell Death Dis. 2018 May 22;9(6):584. doi: 10.1038/s41419-018-0652-4 (PMC5964221; doi:10.1038/s41419-018-0652-4)

## Slide 1
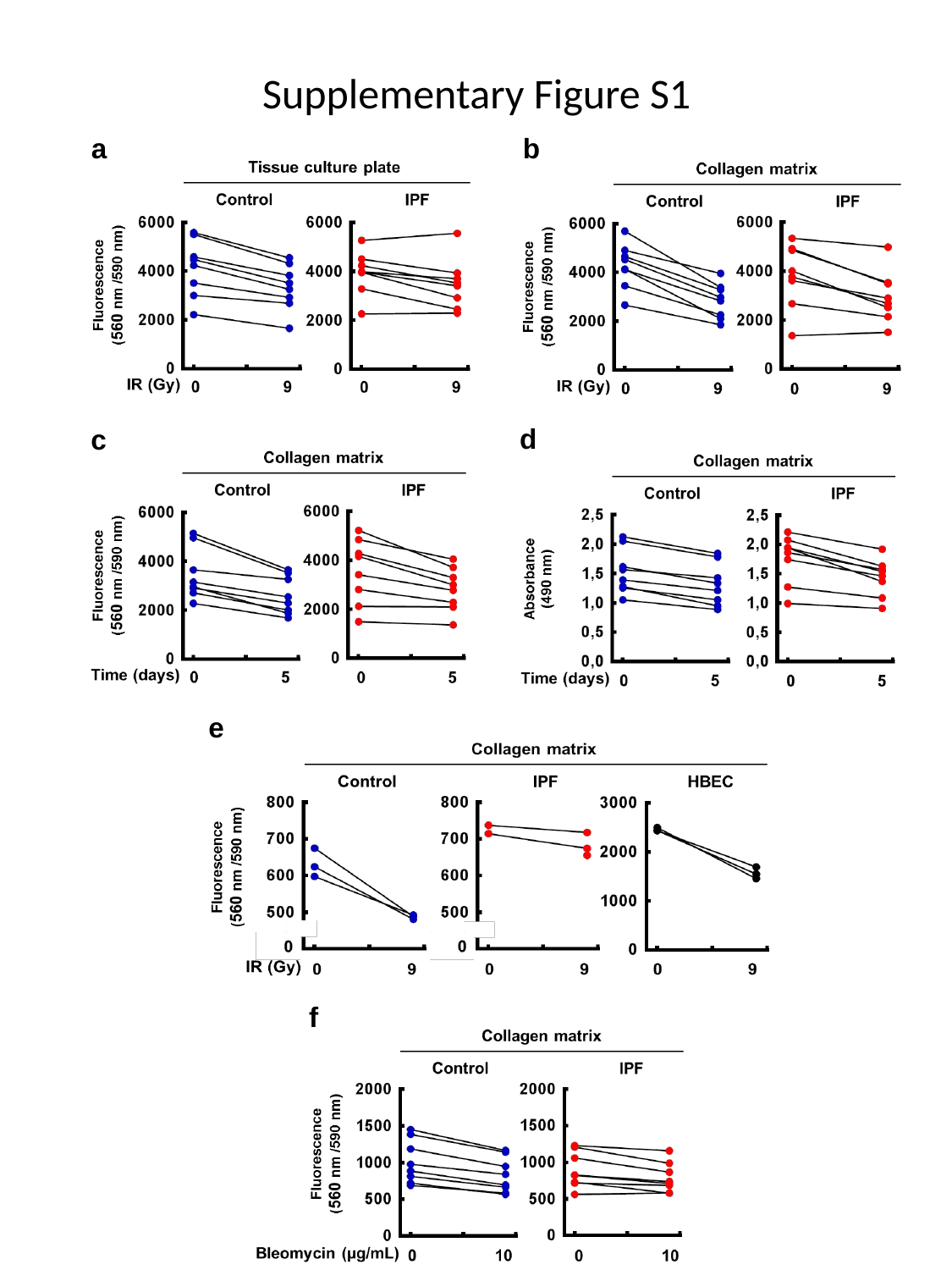

Supplementary Figure S1
b
a
d
c
e
f

Supplement: Supplementary file 1 — Supplementary Figure 1 [file 41419_2018_652_MOESM1_ESM.pptx]

## Slide 1
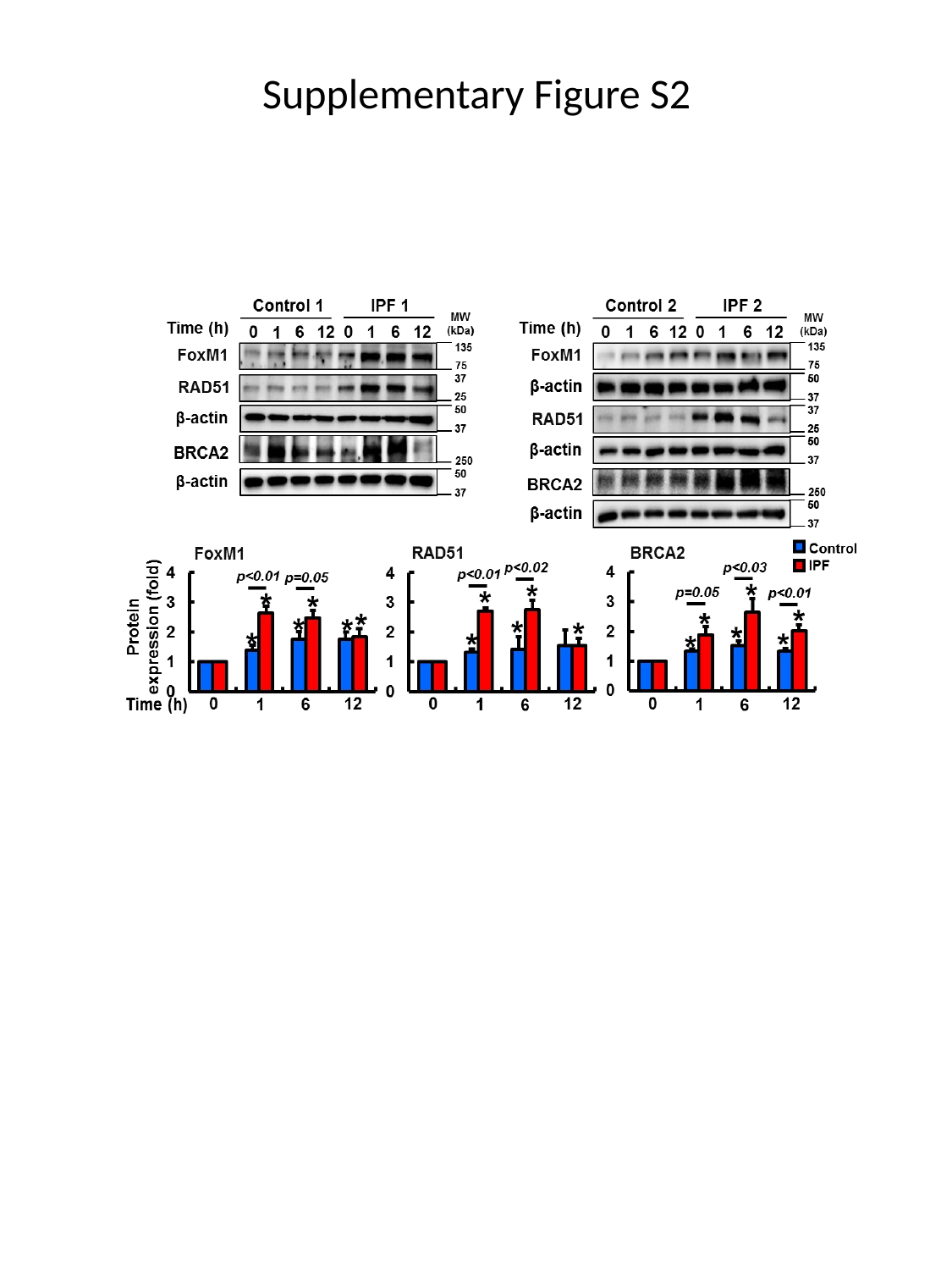

Supplementary Figure S2

Supplement: Supplementary file 2 — Supplementary Figure 2 [file 41419_2018_652_MOESM2_ESM.pptx]

## Slide 1
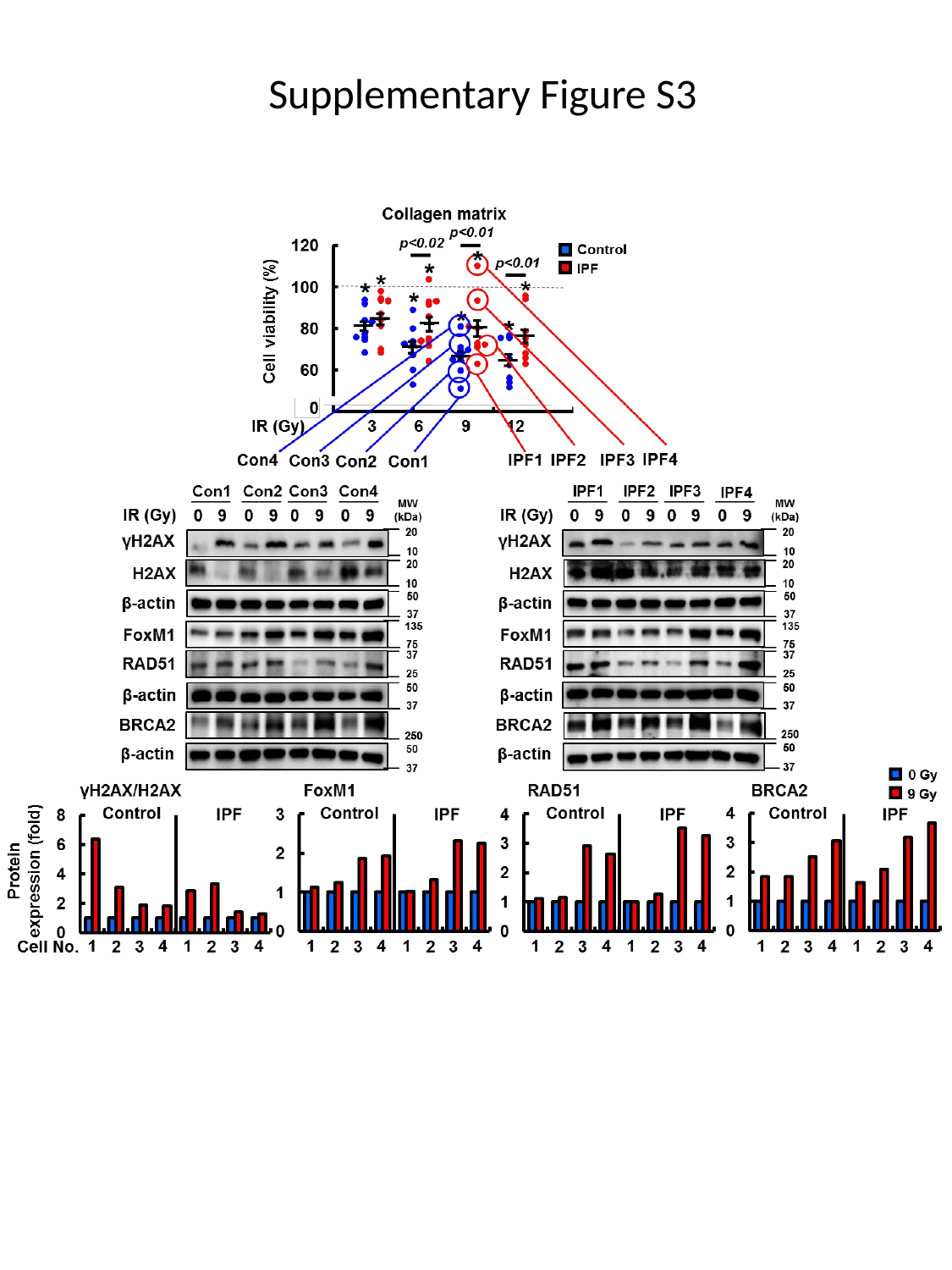

Supplementary Figure S3

Supplement: Supplementary file 3 — Supplementary Figure 3 [file 41419_2018_652_MOESM3_ESM.pptx]

## Slide 1
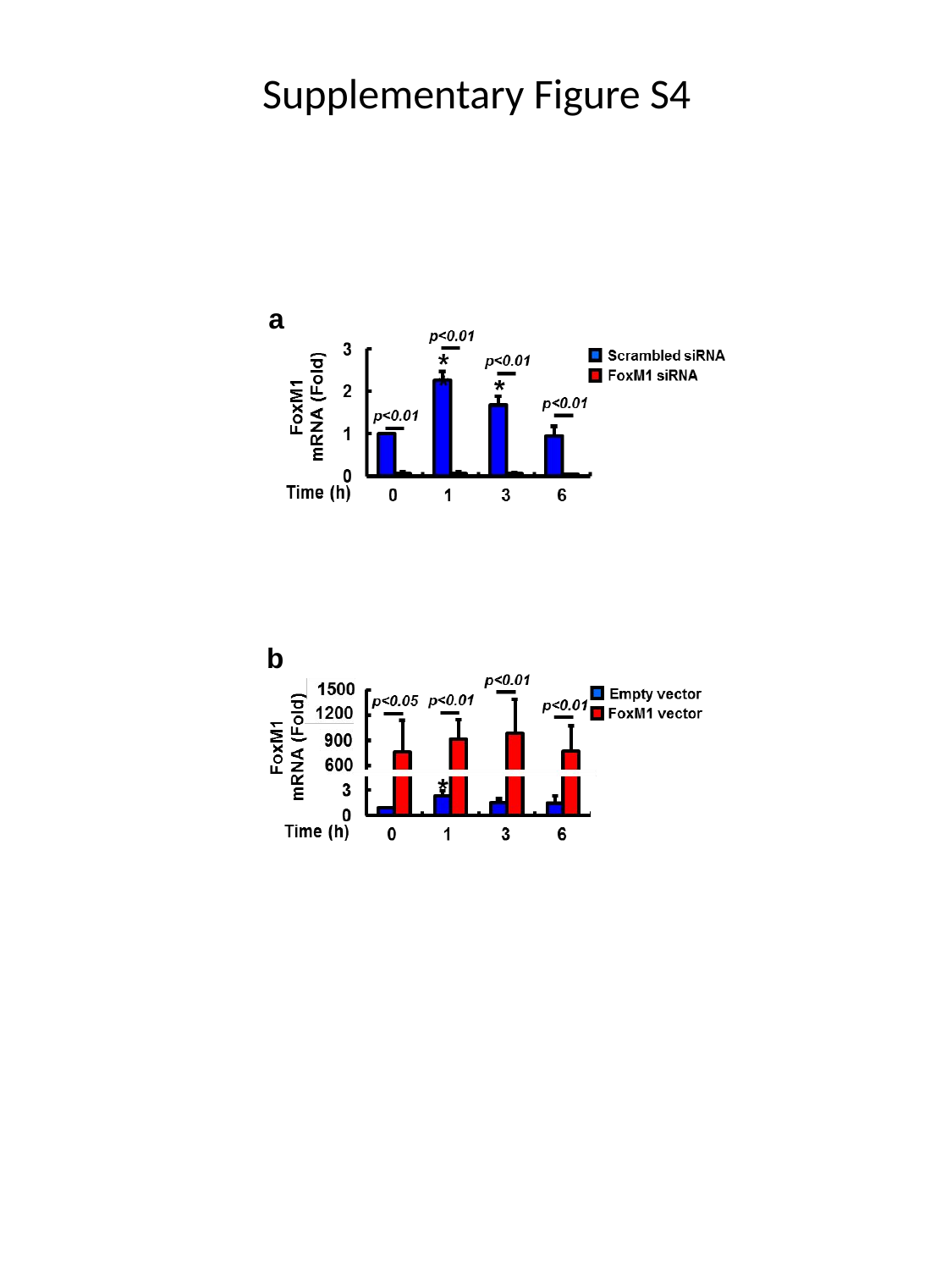

Supplementary Figure S4
a
b

Supplement: Supplementary file 4 — Supplementary Figure 4 [file 41419_2018_652_MOESM4_ESM.pptx]

## Slide 1
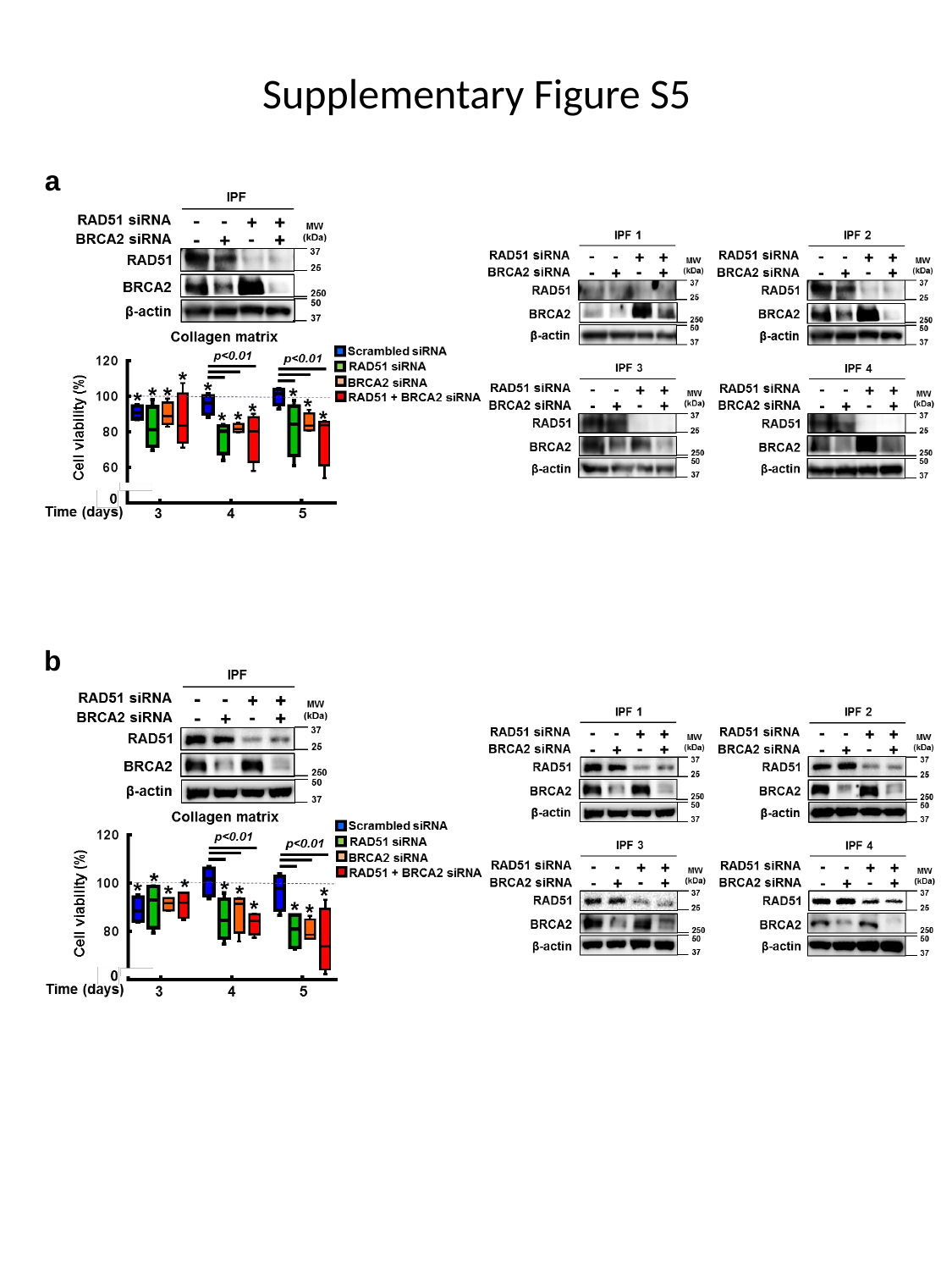

Supplementary Figure S5
a
b

Supplement: Supplementary file 5 — Supplementary Figure 5 [file 41419_2018_652_MOESM5_ESM.pptx]
