# Supplementary material for: FoxM1-dependent RAD51 and BRCA2 signaling protects idiopathic pulmonary fibrosis fibroblasts from radiation-induced cell death
Source: Cell Death Dis. 2018 May 22;9(6):584. doi: 10.1038/s41419-018-0652-4 (PMC5964221; doi:10.1038/s41419-018-0652-4)
Supplement: Supplementary file 6 — Supplementary figure legends [file 41419_2018_652_MOESM6_ESM.docx]

**Supplementary Figure S1** **IPF fibroblasts are resistant to genotoxic insult.** Cell viability at 5 days following radiation (9 Gy) was measured for each control and IPF fibroblast (n=8, each) on a tissue culture plate (**a)** or a collagen matrix (**b and c**). **c** Cell viability was serially measured over 5 days following radiation (9 Gy). Fibroblast viability was measured at 560/590 nm as described in the Materials and Methods. **d** Each control and IPF fibroblast was irradiated at 9 Gy, and their proliferation was measured at 490 nm on collagen as described in the Materials and Methods. **e** Viability for control, IPF fibroblast and HBEC cells was measured at 5 days following radiation (9 Gy) on collagen and compared to unirradiated (0 Gy) cells. **f** Control and IPF fibroblast cultured on collagen were treated with bleomycin (10 µg/ml). Fibroblast viability was then measured after 24 h and compared to untreated (0 µg/ml) cells.

**Supplementary Figure S2 FoxM1 and its target RAD51 and BRCA2 are increased in IPF fibroblasts treated with bleomycin.** Control and IPF fibroblasts attached to collagen matrix were treated with bleomycin (10 µg/ml) for 0-12 h. Protein expression of FoxM1, RAD51, and BRCA2 was then measured. *Upper*, representative FoxM1, RAD51 and BRCA2 protein expression in control and IPF fibroblasts (n=8, each) treated with bleomycin. *Lower*, fold changes of FoxM1, RAD51 and BRCA2 in control and IPF fibroblasts over time. Values are presented in mean ± SEM of fold changes compared to unirradiated control or IPF fibroblasts set at 1 fold.

**Supplementary Figure S3** **FoxM1-dependent DNA repair protein expression in radioresistant control and radiosensitive IPF fibroblasts.** Control fibroblasts that were resistant to radiation induced cell death and radiosensitive IPF fibroblasts shown in Fig. 1b were selected. Cells were cultured on collagen matrix and irradiated (9 Gy). 6 h following radiation, γH2AX, H2AX, FoxM1, RAD51, and BRCA2 levels were then measured. Values are presented in mean ± SEM of fold changes compared to unirradiated control or IPF fibroblasts set at 1 fold.

**Supplementary Figure S4** **Confirmation of loss or gain of FoxM1 mRNA in IPF or control fibroblasts, respectively.** **a** FoxM1 was silenced by FoxM1 siRNA in IPF fibroblasts (n=3). **b** FoxM1 was overexpressed in control fibroblasts (n=3) by FoxM1 vector. FoxM1 mRNA expressions were then examined as a function of time after 9 Gy radiation. Values are presented in mean ± SEM of fold changes compared to unirradiated IPF fibroblasts transfected with scrambled siRNA or control fibroblasts overexpressing empty vector set at 1 fold. *: statistical significance of FoxM1 mRNA expression compared to unirradiated IPF fibroblasts transfected with scrambled siRNA or control fibroblasts overexpressing empty vector at *p*<0.05.

**Supplementary Figure S5 Silencing of RAD51 and/or BRCA2 sensitizes IPF fibroblasts to radiation-induced cell death.** **a and b** *Right*, RAD51 and/or BRCA2 silencing was confirmed by Western analysis. *Left,* the effect of silencing RAD51, BRCA2 or both RAD51 and BRCA2 on IPF (n=4 each) cell viability over time following 9 Gy. Values are presented in mean ± SEM of percentages compared to unirradiated IPF fibroblasts in each experimental group at 100% (dotted line). *: statistical significance of cell viability compared to unirradiated IPF fibroblasts in each experimental group at *p*<0.05. Radioresistant IPF fibroblasts (high viability after 9 Gy) were selected for these experiments.
